# Supplementary material for: Gene Regulatory Network of the Noncoding RNA Qrr5 Involved in the Cytotoxicity of Vibrio parahaemolyticus during Infection
Source: Microorganisms. 2022 Oct 21;10(10):2084. doi: 10.3390/microorganisms10102084 (PMC9610228; doi:10.3390/microorganisms10102084)
Supplement: Supplementary file 1 [file microorganisms-10-02084-s001.zip › microorganisms-1940400-supplementary.pdf]

Gene regulatory network of the noncoding RNA *Qrr5* in the cytotoxicity of *Vibrio parahaemolyticus* during infection

Supplementary materials

Fei Zha[12#], Rui Pang[2#], Shixuan Huang[2], Jumei Zhang[2], Juan Wang[2], Moutong Chen[2], Liang Xue[2], Qinghua Ye[2], Shi Wu[2], Meiyan Yang[2], Qihui Gu[2], Yu Ding[2], Hao Zhang[1*], Qingping Wu[2*]

[1] School of Food Science and Technology, Jiangnan University, Wuxi 214122, Jiangsu, China

[2]Guangdong Provincial Key Laboratory of Microbial Safety and Health, Ministry of Agriculture and Rural Affairs, Key Laboratory of Agricultural Microbiomics and Precision Application, State Key Laboratory of Applied Microbiology Southern China, Institute of Microbiology, Guangdong Academy of Sciences, Guangzhou 510070, Guangdong, China

#These authors have contributed equally to this work.

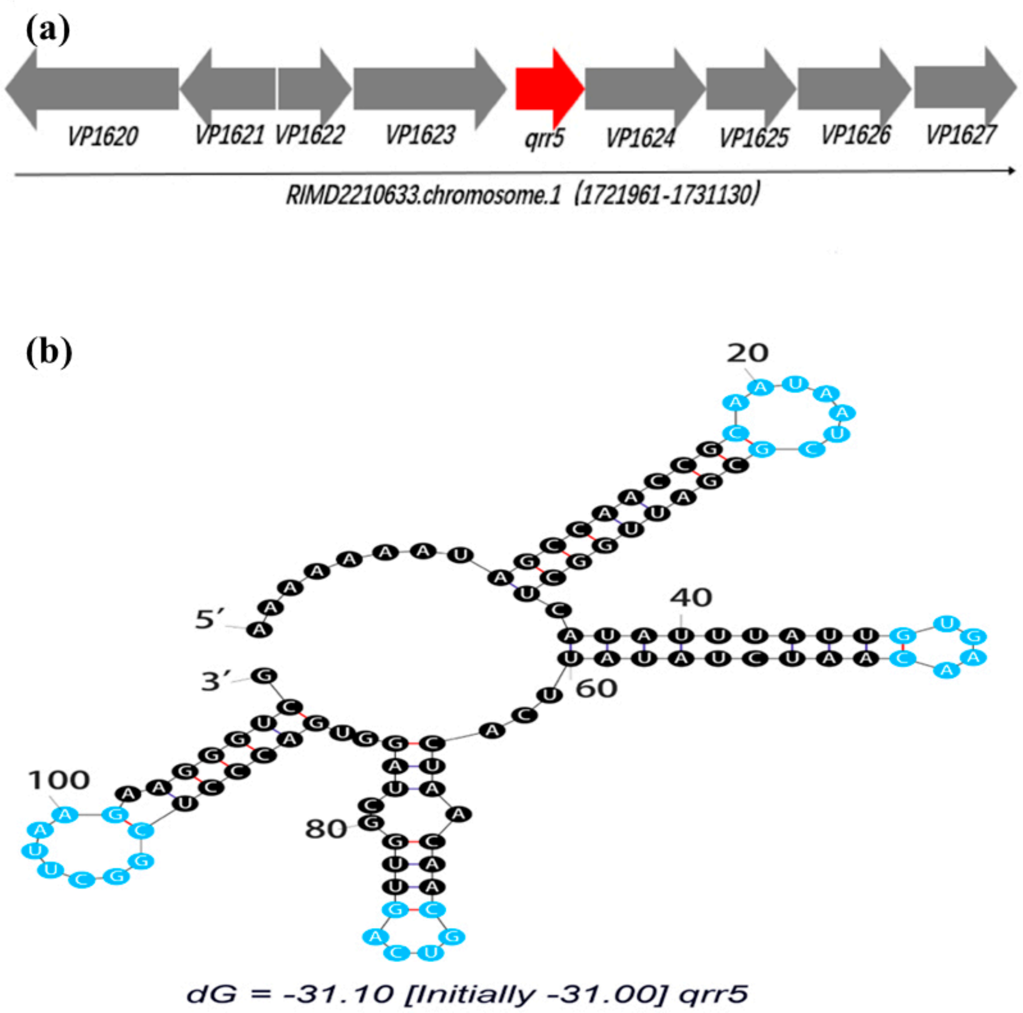

Figure S1. The genetic map and structure of sRNA *Qrr5* in *V. parahaemolyticus*.

(a) The position of *Qrr5* in the RIMD2210633 chromosome 1. (b) The secondary structure of *Qrr5* (109 bp) in *V. parahaemolyticus*.

Table S1. Oligonucleotide Primers

Primer	Sequence (5'—3')
For the construction of the *Qrr5*-deletion mutant	
Qrr5-U-F	CTTCTAGAGGTACCGCATGCGCTGCTAAAAGAAG
Qrr5-U-R	ATGCATAATACTTCTGCCTGTCGTATATGA
Qrr5-D-F	TCATATACGACAGGCAGAAGTATTATGCAT
Qrr5-D-R	GTGGAATTCCCGGGAGAGCTGTGATCAGCTTCGCTAAGG
Qrr5-F100	AAGTGAAGCGCGCCGTACCAACCTCATTACCGAT
Qrr5-R100	ATCGCGTTTCTCTACCTGAC
Qrr5-pF	GCTCTAGAGTGAGGGAAGTGCAATTTGC
Qrr5-pR	CCTCGAGCGACCCTTCTTAAGCCGAG
For quantitative PCR	
16s-F	AAGCGTGGGGAGCAAACAG
16-R	CGAAGGCACCAATCCATCTC
qrr1-F	CGACCCCTCGGGTCACCTAG
qrr1-R	AAAAAAGAAGCCAATAGGCAGTCGG
qrr2-F	GACCCTTATTAAGCCGAGGG

qrr2-R	AGAAAAAACGCCAATCACA
qrr3-F	TGACCCTTTCTAAGCCGAG
qrr3-R	AAAAAAAAAGCCAACCACAAGG
qrr4-F	CGAGGGTCACCTAGCCAACT
qrr4-R	AAATAGCCAACCGCAAAGAG
qrr5-F	AAAAAAATAGCCAACCGCAA
qrr5-R	CGACCCTTCTTAAGCCG
VP1766-F	AAACAATGCGTGAGCCAGAC
VP1766-R	ACCAAACAGAACTCACCTCCAC
VPA1068-F	GAAAACACCCGCCAACATTAC
VPA1068-R	TCGCACTTCGGCATCATTG
VP2492-F	GAACAGCAAGAATGACCAAAGC
VP2492-R	TGTACGTCGATAACGGAGAAGG
VP0485-F	GCAGTACGATGGCGAAAGC
VP0485-R	ACACCAAAGCGGGGACAA
VP2914-F	ATTTGCACAAACCTTCCACATC
VP2914-R	GAGCAACAAGCTGGCTTCATC
VP0486-F	CAAGCGAACCTTGAACTTGAGA
VP0486-R	GCCCGTAACAGTCGTTGTAATC
VPA0628-F	CGGTTGTGATTGCACTGGG
VPA0628-R	TCGCCTTTTGGCAGATGAG
VP1665-F	TGTTCGAGTGTCGGAAGCG

VP1665-R	ATCAGTCGGGCGAGCGTAT

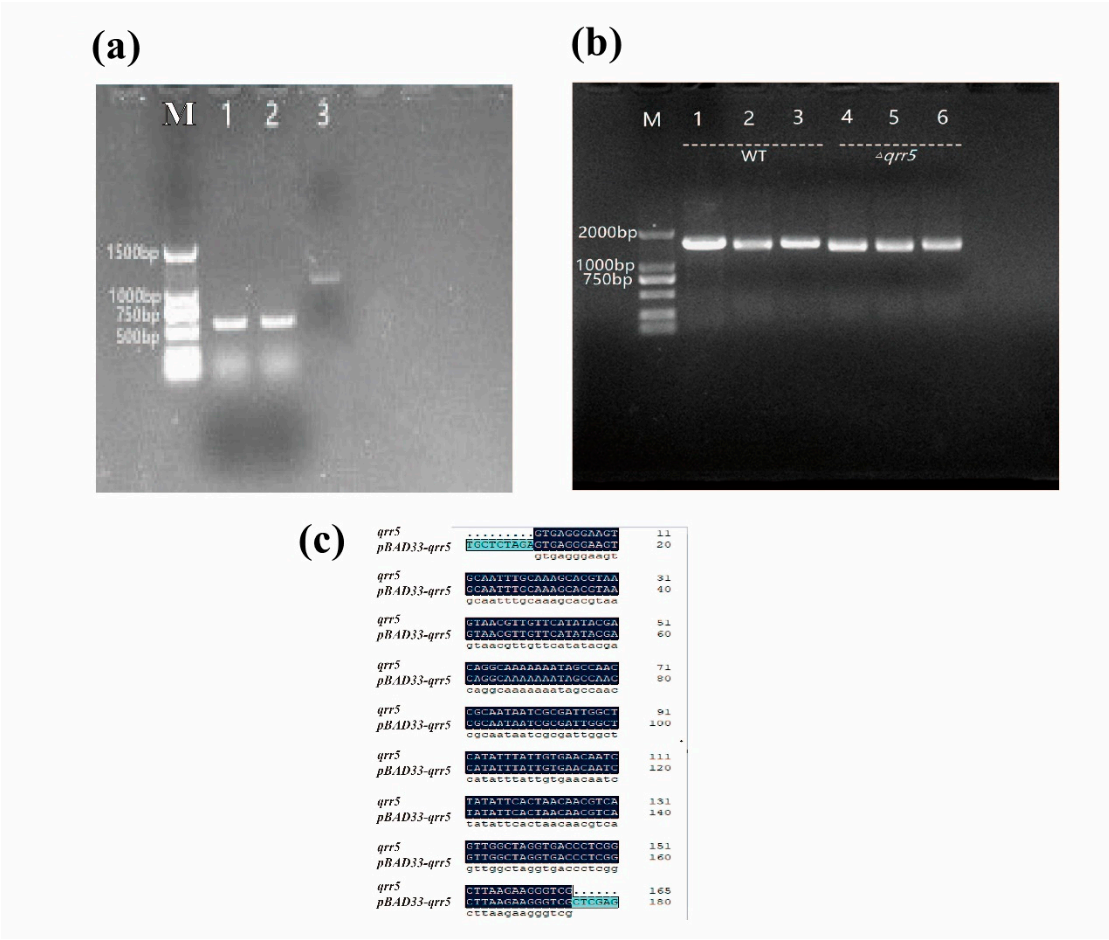

Figure S2. Construction and confirmation of *ΔQrr5*. (a) M: DNA marker; 1: The upstream DNA fragment (600 bp) of *Qrr5* was amplified by PCR with primers *Qrr5*-U-F/*Qrr5*-U-R; 2: The downstream DNA fragment (600 bp) of *Qrr5* was amplified by PCR with primers *Qrr5*-D-F/*Qrr5*-D-R; 3: The confused DNA fragment of upstream and downstream (1200 bp). (b) M: DNA marker; 1-3: the DNA fragments (1509 bp) of upstream-100bp to downstream +100 bp of *Qrr5* (109 bp) were amplified by PCR with primers *Qrr5*-F100/*Qrr5*-R100 using the *V.parahaemolyticus* wild-type chromosomal DNA as templates; 4-6: The DNA fragments (1400 bp) of upstream -100 bp to downstream +100 bp of *Qrr5* (109 bp) was amplify by PCR with primers *Qrr5*-F100/*Qrr5*-R100 using the *V.parahaemolyticusΔQrr5* chromosomal DNA as templates, respectively. (c) The sequence of DNA fragment (109 bp) of *Qrr5* was amplified by

PCR with primers *Qrr5*-pF/*Qrr5*-pR using the *V.parahaemolyticusΔQrr5::pBAD33-Qrr5* chromosomal DNA as templates compared to the sequence of *Qrr5*.

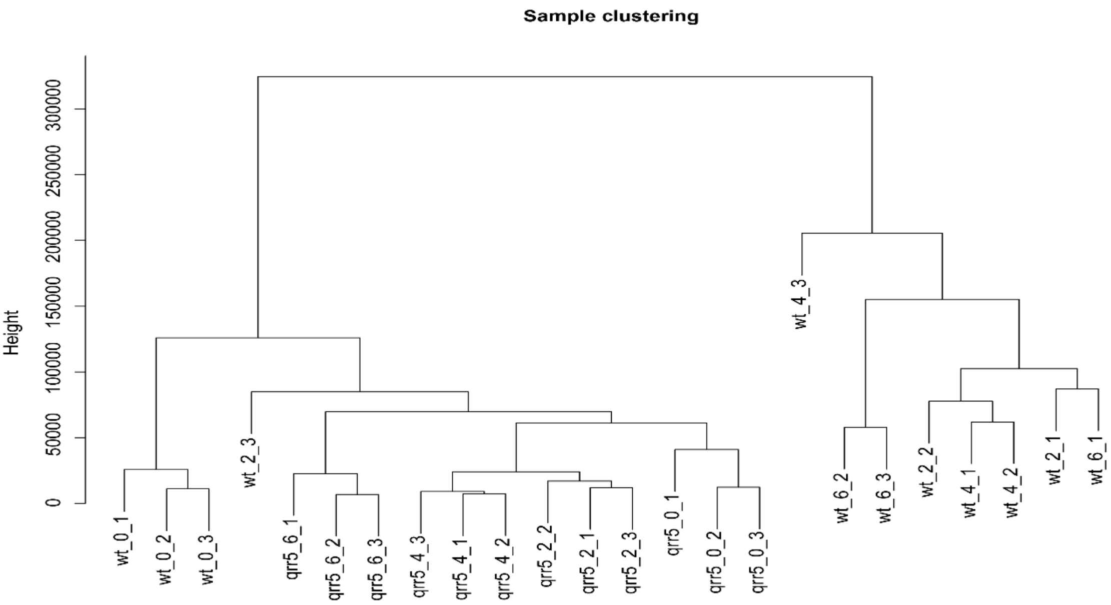

Figure S3. Each sample clustering of RNA-Seq data.

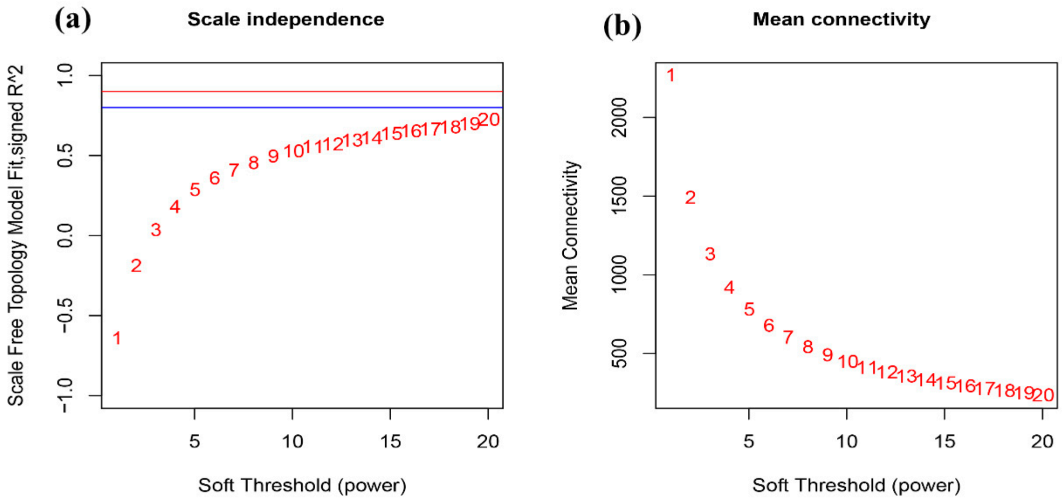

Figure S4. The scale independence (a) and mean connectivity (b) of RNA-Seq data.

Table S2. The function of genes in the WGCNA co-expression network

Name	Function
VP1766	Alanyl-tRNA editing protein
VP1600	Unknown
VPA1068	Hypothetical protein
VP2914	D-2-hydroxyacid dehydrogenase
VP0027	Hypothetical protein
VP0807	Unknown
VP0486	Diguanylate cyclase
VPA1520	Hypothetical protein
VP2389	Magnesium transporter
VP2492	Ammonium transporter
VP2112	Unknown
VPA0631	Heme synthase (cyoE)
VPA1141	Carboxymuconolactone decarboxylase
VP0485	TIGR01212 family radical SAM protein
VPA0628	Cytochrome ubiquinol oxidase subunit I (cyoB)
VP1665	Type III secretion I chaperone SycN
VP2012	Unknown

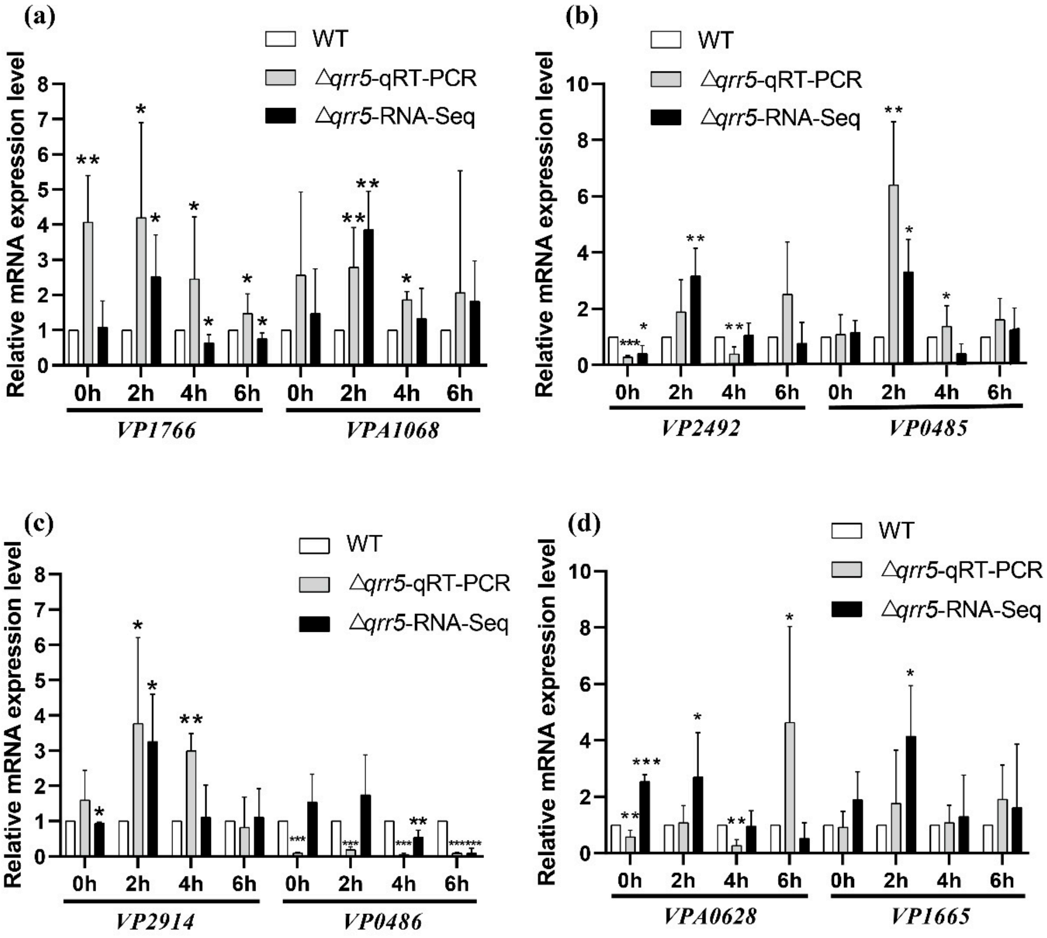

Figure S5. Relative expression of several differentially expressed genes (*ΔQrr5*/WT) at the mRNA level determined by qPCR during various infection periods. (a) The expression of *VP1766* and *VPA1068* by qRT-PCR analysis. (b) The expression of *VP2492* and *VP0485* by qRT-PCR analysis. (c) The expression of *VP2914* and *VP0486* by qRT-PCR analysis. (d) The expression of *VPA0628* and *VP1665* by qRT-PCR analysis.
